# On the Legal Responsibility of Females during Pregnancy and Parturition

**Published:** 1839-01

**Authors:** 


### Art. VI.

*Die Zurechnungsfähigkeit der Schwangern und Gebärenden beleuchtet* Von Dr. Johann. Chr. Gottfr. Jörg, ordentlichem Professor der Geburtshülfe an der Universität zu Leipzig, &c. &c.

*On the Legal Responsibility of Females during Pregnancy and Parturition.* By Dr. J. C. G. Jörg, Professor of Midwifery in the University of Leipzig.—*Leipzig*, 1837. pp. 419.

The title of this work is calculated to convey but a faint idea of the number and importance of the subjects of which it treats. The development of the generative functions in the two sexes, the influence which this development exerts upon the body, the changes induced by pregnancy in the female, the signs and symptoms of that state, the growth of the fœtus, the natural and physical causes of abortion, the doctrine of protracted gestation, the psychological changes which take place in the female at the period of parturition, and the accidental and natural causes of the death of the new-born child, are questions which are here fully discussed. We proceed at once to offer an analytical digest of some of the more important of these subjects to the notice of our readers, and endeavour to show their application to the principles and practice of medical jurisprudence.

The volume commences with some introductory remarks on the sexual functions, and on the dangers which result from the abuses of these in either sex. We find here but little calling for particular notice. The author considers that, at the age of puberty, there is a greater tendency for the individuals of the two sexes to be guilty of acts not only inconsistent with their usual conduct, but often leading to serious criminal responsibility. He explains this by supposing a sympathetic reaction to be exerted through the sudden development of the generative organs on the brain. Without entirely adopting this view, it certainly has often struck us as a very remarkable fact that, in our own agricultural districts, so many young females, about the age of puberty, should have been guilty of acts of incendiarism; acts often perpetrated without the least apparent motive, and against their best friends. On these occasions, unfortunately for the accused, it has not been thought necessary to examine into the state of mind of the party: the *sudden* manifestation of insane conduct in females so situated not being as yet considered, by legal authorities, a good ground of defence.

That unmarried females who have conceived are much more likely to have still-born children than those who have become impregnated in the married state, is well known. Sorrow, anxiety, shame, and an overwhelming mental depression at her condition, sufficiently explain why the unmarried woman should be exposed to greater risk during pregnancy and parturition than the married. This is a question which it is by no means unimportant for the medical jurist to bear in mind, since, among the unmarried, lie in general the greater number of charges of infanticide. We have already, in a previous number, (No. VII. p. 235,) alluded to this subject; and have there given some statistical tables relative to still-births. We are glad to be able to add to these the observations of Professor Jörg, in Leipzig, during the years 1835-6.

"In 1835 there were born 1131 legitimate and 249 illegitimate children. Of the former 45, and of the latter 28, were still-born. In the year 1836, there were 1135 legitimate and 242 illegitimate births. Of the former 52, and of the latter 18, were still-born. In the first year the number of illegitimate still-births was at its maximum, in the second year at its minimum degree; for, in 1835, one illegitimate child was still-born in $8\frac{25}{28}$ births; and, in 1836, one in $13\frac{8}{18}$. The ratio of still-born among legitimate births was, for 1835, one in $25\frac{6}{45}$; and, in 1836, one in $21\frac{43}{52}$." (p. 22.)

It will be seen that these conclusions very closely approximate to the numbers given in the tables to which we have already alluded.

Dr. Jörg has divided his work into two parts. In the first he treats of responsibility in the *pregnant* state, and in the second of responsibility as it affects a female during *parturition*. In the outset he does not hesitate to announce that his object is to put forward all the resources of science in the defence of unmarried females, who may, while thus situated, become the subjects of criminal charges: "This book," he observes, "is intended to show how far the resources of obstetric sciences are adapted to set aside accusations made without sufficient grounds against unmarried females during pregnancy and parturition, or to diminish or entirely remove responsibility for their acts." (p. 44.)

In the second chapter the author proposes the question: Whether a female can become conscious of her pregnancy from her own feelings without communication with others? This question, put in various forms, has received much attention from medical jurists, and has been differently answered. Conception, as it is well known, takes place independent of the will both of the male and female; and, although certain feelings in the female have been said to indicate conception, yet the best informed observers put no trust in these. Women often become pregnant without being in the least aware of their condition; and several months may pass before they are convinced by their feelings, that they are actually pregnant. The frequent mistakes made by females in married life, and by those who have borne children, show how difficult it is for a woman to know whether or not she be really pregnant; and, *à fortiori*, the difficulty will be greater where the female is unmarried, and has become so for the first time. It is not our intention to follow the author through his examination of the signs of pregnancy: this is the less necessary, since a recent examination of the work of Dr. Montgomery on this subject has enabled us to lay before our readers a summary of the best modern opinions on the changes induced by the pregnant state. It has often been laid down as a medico-legal axiom, that a woman who has exposed herself to the causes of conception, ought to be aware of, or at least prepared for her pregnancy. Jörg combats this opinion; because, he says, "it can never imply a *certainty* of knowledge, on the part of the woman, while many of the criminal charges brought against her rest upon her having a certain knowledge of her condition." (p. 44-45.)

In speaking of the signs of pregnancy, the author does not seem to be aware of the observations of Dr. Montgomery, relative to the changes in the colour and development of the areola around the nipples. The swelling of the breasts and the secretion of the milk are the only conditions to which he looks, contending that diseases of the uterus and ovaries may give rise to similar changes. The symptoms of quickening and the subsequent motions of the child, are also dismissed as in themselves inconclusive. Numerous morbid causes may lead to a suspicion of quickening, while the motions of the child may from position and other circumstances be unperceived or unnoticed. Auscultation is a most excellent aid for the detection of pregnancy; but this applies only to its use in the hands of medical men. A woman who is unable to discover that she is pregnant by her feelings is of course unable to acquire any knowledge of her condition by her ear. In relation to the question proposed, our author after having examined every sign of pregnancy as it affects a female, comes to the conclusion that "a woman may for a very long period, even until the access of parturition, be unable to acquire from her own feelings any *certain knowledge* of her pregnancy." (p. 79.) He then recommends the adoption of some plan by which unmarried females, who are pregnant and advanced to the seventh or eighth month of gestation, may be induced to submit to medical examination and have their wants provided for. A system of espionage on the part of midwives officially appointed might, he thinks, be adopted with advantage. The author gravely adds:

"If an unmarried female, whose *external appearance* (!) leads to a suspicion of pregnancy should refuse to submit to an examination by the appointed midwife, the law ought to arm the latter with sufficient power to enforce it; since the only possible way of ascertaining whether a woman be or be not pregnant, is to make her undergo an examination by an experienced person." (p. 83.)

This plan, it appears to us, comes somewhat singularly from one who has just been using the strongest arguments to prove the uncertainty of the outward signs of pregnancy. A midwife in a village or town is to judge from *external appearance* what unmarried females may be pregnant or not; and the law is to compel them to submit to an examination under the dictation of a woman, who may, without any great imputation on her acuteness, be mistaken in nine cases out of ten! Dr. Jörg seems to have forgotten what is a notorious fact, that unmarried females who are pregnant, take every means to conceal external appearances; and so effectually do they succeed in this, that they are often actually delivered in the houses of persons who have seen them daily at their work without entertaining the least suspicion of their being pregnant. Besides, even were this not the case, numerous diseases in unmarried females put on the external appearance of pregnancy; it does not, however, appear that any rules are laid down to enable the midwife to distinguish a prominence of the abdomen depending on morbid causes, from one due to the presence of a fœtus in the womb!

Most medical jurists will agree with Jörg, that a pregnant unmarried female may remain for a long period ignorant of her condition; and, indeed, that in some instances she may acquire no *certain knowledge* of it until the time for parturition approaches. Many unmarried females, however, notwithstanding that ignorance which Jörg alleges to exist among them, will have good reasons to suspect their condition long before the termination of their pregnancy; and if even suspicion existed, this would assuredly lead a woman, whose intentions towards her offspring were innocent, to prepare for what common sense would show to be a natural contingency. We cannot join the author in thinking, that before criminal charges are urged against unmarried females, it should be proved that they had a *positive knowledge* of their actual state. Many females in married life, even with the aid of an accoucheur, do not always acquire this certainty of knowledge until parturition commences. It is absurd then to contend for a kind of proof, not only, morally speaking, unnecessary, but medically impossible to attain. Indeed, it is tantamount to affirming, that criminal designs ought never to be imputed to women thus situated; a doctrine which, however humane, is inconsistent with the rules of civilized society.

The physical and moral changes induced in the female system by pregnancy are next considered. The singular desires and feelings which sometimes manifest themselves in pregnant women are portrayed, and their origin ascribed to the sympathetic stimulus exerted on remote organs by the enlargement of the uterus and development of its contents. It is not our purpose to dwell on these, nor on the presentiments of misfortune or death which so often beset females under these circumstances. In relation to the question, how far responsibility is diminished by *these changes* in body and mind he observes:

"That in some instances they may be such as to mitigate, and in others entirely to remove responsibility. The oppression of mind and body which exists in married females when pregnant, will be aggravated in the unmarried woman's by remorse, shame, the fear of ill-treatment, the consciousness of poverty, and abandonment by her seducer. Under such affliction, the mind may become so disturbed as to lead to the perpetration of some immoral or unlawful act, for which we may find palliation when we can trace it to the operation of fear or irrepressible melancholy. In fits of syncope, asphyxia, and convulsions, to which pregnant women are subject, all consciousness is lost, and responsibility for their acts must here cease. A serious question might arise, as to how long before or after the attack the free exercise of reason was suspended, more especially when a woman has borne a child while suffering under any of these conditions. This is a case in which a charge of concealed birth or of wilful abortion might be raised against the woman, and one which she would not have it in her power easily to rebut." (p. 116, *et seq.*)

We consider that the author lays too much stress upon the psychological changes induced by pregnancy. The desires of pregnant women are commonly of a very harmless nature; they do not manifest themselves by acts of murder, incendiarism, or other crimes endangering the peace of society. In the unmarried we but too often see the morbid melancholy leading to the commission of suicide, a circumstance to which Dr. Jörg does not refer; his object being to exculpate these unfortunate beings from crimes committed against others, a case which is however so rare, as not within our knowledge to have called for the assistance of a medical jurist. We of course except the crime of infanticide, which refers to the *parturient* and not to the pregnant state.

The fourth chapter introduces us to a curious disquisition upon how far human attributes and human rights apply to the fœtus in utero. Dr. Jörg complains that "neither medical nor legal writers have sufficiently considered the peculiar and circumscribed life enjoyed by the embryo, nor the wide difference which exists between the born and the unborn child." After having traced the development of the embryo, and shown how closely it is dependent on the mother for continued existence and growth, he candidly states his opinion that the human fœtus is to be regarded only as a higher species of *intestinal worm* (p. 138 and 146); that while in the uterus it is not endowed with a human soul nor is it entitled to human attributes! (p. 148.) The contrary opinion is set down as a wild fiction, taken up, it is true, by the majority of jurists and physicians, but without sufficient grounds, and contradicted by the whole history of the uterine development of the child.

This singular doctrine, it will be remarked, is not far removed from that of the ancient Stoics, who regarded the child as only acquiring a soul and the rights of a human being when it had performed the act of respiration. The fœtus in utero was treated as *pars viscerum matris.* A natural result of this opinion, was, that wilful abortion could not be regarded as a crime; and that it was just as innocent a matter to expel this intestinal incumbrance by artificial means as if the question related to the expulsion of the contents of the bowels. We do not find that Dr. Jörg carries his views exactly to this extent; but the inference is plain: if his views be correct, wilful abortion is neither criminal nor immoral, and the statute books of all civilized countries are disgraced by a law pregnant with injustice. We shall not, however, allow him to escape by an evasion of this kind; for the real question is not how far he himself chooses to carry his doctrine, but to what extent it may be legitimately carried. That he has anticipated considerable opposition to his opinions on this subject is plainly expressed in the preface, where he begs those of his readers, who in spite of his reasoning still hold to what he regards as a prejudiced view, "to examine in some anatomical museum the ovum with its membranes, and say whether from its appearance it has a claim to be considered a human being." (ix.)

Among the reasons for the adoption of this opinion we find the following set forth by the author, "that there is no direct sanguineous communication between the mother and child (p. 120); that the abdomen of a female is too confined a cavity for a human being to be formed in; that its organs are imperfect and only adapted for a future use; that it enjoys only the restricted life of a water-worm (p. 126); that the membranes in which it is placed constitute an essential part of it; and that human attributes, if applied to the child, ought equally to be applied to the membranes without which its life cannot continue (p. 127-143;) that, while in the uterus, it only receives chyle and oxygen through the blood of the mother; that, from its situation as well as its low and defective organization, it cannot receive any mental impression; that, of all the senses, taste and feeling are the only two which it can be considered to possess, and these in an imperfect degree. (p. 140.)"

We find here some facts enumerated well known to physiologists, and proving nothing more than that the development of a human being is a slow and gradual process, and consists of a succession of stages, between any two of which it is impossible to draw a line of demarcation. We cannot see how the rights of humanity in a child should be made to depend upon whether there be or be not a distinct anastomosis between the blood-vessels of the uterine and fœtal portions of the placenta! We do not understand what is intended by the assertion that the female abdomen is too *confined* a space for the formation of a human being; since human rights are not regulated by the size or weight of a human being. When the argument is made to rest upon the fact of the fœtal organs being *imperfect* and adapted only to a *future* use, we must beg the author to remember that male and female children are born with sexual organs which possibly, at birth, he would pronounce to be as imperfect in regard to the generative function as the lungs of the unborn child are in regard to the function of respiration. The organs are destined for a future use, but what has this to do with the rights of humanity? The placenta and membranes, because necessary to the existence of the fœtus, are held to be a substantive part of the being, and entitled to the same attributes; but this is a piece of sophistry which would easily be exposed by a tyro in physiology. The placenta and membranes are the media through which the unborn child derives existence, as air and food are the media through which the life of the born child is supported. A child will die if the connexion with the placenta be destroyed, just as an adult will perish when the access of air and food is cut off: but because these are undeniable facts, are we to confound the media through which existence is supported with human existence itself? The answer is one as much of common sense as of physiology; and no sound moralist or legislator has hitherto thought of denying or granting human rights, according to whether the being derived its support through the blood of its mother or through the air and food received into its body after birth. Lastly, it follows, if because the senses are shut up and there are no mental impressions while the child is in utero, it is not to be regarded as a human being, Professor Jörg would only be consistent in excluding from this title all those children that are born idiots or deaf and blind.

We should not have occupied our space in exposing such palpable fallacies, were it not that Professor Jörg is a man who has earned in some sort, and deservedly, a European reputation; and whose opinions are therefore likely to be diffused and adopted by a certain class of readers.

We shall now take the liberty of stating why the most eminent jurists, legislators, and physicians, hold an entirely different opinion to that which he has chosen to adopt; and why in all civilized countries such an opinion is made the basis of legislation in respect to wilful abortion. A regard to the perpetuation of the species, and to the dangerous consequences which so frequently fall on the mother, has induced legislators to visit wilful abortion with punishment, and to endeavour to check it as a crime. These reasons we may consider to be certainly sufficiently strong for legislative interference, whether we regard the child in utero as a human being or not: if jurists in general adopt the former view, it is not, as the author supposes, merely because the fœtus will afterwards become a human being, but because neither reason nor justice will allow them to restrict or limit the period at which a child should acquire the legal rights belonging to its species. He, somewhat superfluously as it appears to us, combats this presumption, by asserting that numerous fœtuses do not live to assume the rights of humanity, but die before or during the act of birth, while many others are born deformed and misshapen. The law has sufficiently provided for these cases; but, in the mean time, it will not consent to treat the child in utero as not human, because all children are not born alive, nor as incapable of inheriting or of having property assigned to it, because some children are born deformed. A *human being*, according to Jörg, is that which is born or has breathed: but most legislators hold, and we consider rightly, that the child *immediately before birth* is as much an object of protection by the laws of society as one which is actually *born.* In either case the being is dependent for the continuance of its existence not upon its own exertions, but upon support derived from extrinsic sources. The difference between a child immediately *before* and *after* birth, upon which the author lays so much stress as a point in which all jurists err, is not to our apprehension greater than that which we see between a child at birth and an adult man, whose mental and bodily powers are in the fullest degree developed. If then this be admitted, where are we to draw the pretended line of distinction between the unborn child at the ninth month and the embryo? It cannot be done. Uterine life, like extra-uterine life, consists of an infinite series of stages so closely allied that no two will admit of separation: nor is it correct to assert that respiration constitutes the boundary between uterine and extra-uterine life; for a child may breathe and die before it is born, or it may be born, and live, and die without respiring. Cases of this description must, we think, strikingly show the fallacy of the author's hypothesis for the settlement of human rights; and we are surprised that it did not occur to him as necessary, before laying down such an arbitrary distinction as he has done, to prove that a new-born child has stronger claims to the protection of the laws than that same child *immediately* before its birth.

We now come to an account of the subject of Abortion, which we consider to be very ably treated. Those abnormal conditions of the female system giving rise to an expulsion of the contents of the womb, especially at the later stages of gestation, are fully exposed; and we need hardly remind our readers how important it is, in a medico-legal view, to be able to appreciate these; since in most cases of unmarried females the pregnancy is concealed, and the abortion, for obvious reasons, a woman so situated will always endeavour to conceal; facts which might be held by the law as affording strong circumstantial proofs of guilt. Sound medical data must here be brought to the exculpation of an innocent woman. The author does not fail to point out that unmarried females are, from the sorrow and anxiety with which they are oppressed, more subject to abortion than the married. The action of popular abortives is then considered: most of these substances are set down as inert, or acting with such violence on the female as to cause the expulsion of the child only at the hazard of her life. The proofs of abortion in the living or dead are attended with more difficulty than is commonly supposed. It is not often that absolute legal certainty on the point can be acquired; and even when the fact of abortion is established, it remains to be shown whether it arose from criminal, accidental, or morbid causes. An examination of the child sometimes affords stronger evidence than that directed to the person of the mother, as the presence of wounds or contusions on its limbs or body. It ought, however, to be clearly ascertained whether the wounds, if present, were inflicted before or after death; a subject on which some sound practical remarks are offered.

After the avowal of his opinions respecting the condition of the fœtus, we are not surprised to find Professor Jörg announcing some loose notions regarding the laws of different countries as they affect criminal abortion. He appears to consider it a crime rather against the health of the mother than against the fœtus, and exculpates unmarried females who make the attempt on themselves, on the ground "that they may not be aware of their pregnancy, and may have taken the abortives merely to rid themselves of their uneasiness: that they know nothing about the life or organization of the fœtus; and that they are unconscious while adopting these abortive measures, that they are actually destroying the life of a human being." If, however, as gestation advances, "a woman becomes convinced of her pregnancy, how is she to be aware that the child within her womb is *regularly organized?* Is it not possible that the abortive means may have been actually employed by her against some indurated ovum, or some diseased or monstrous fœtus, incapable of surviving if it were actually born? Besides, who is to say that the child may not have died from the effects of fear and sorrow in the mother before the abortive means began to operate?" (p. 224.) Such are the loose justifications offered by Professor Jörg for the crime of abortion. We shall make no further remark than to say, that on the one hand his hypothesis, and on the other his humane feelings, have carried him much farther than we imagine most reasonable persons will be inclined to follow him.

The seventh chapter is full of interest, touching upon the question of the length of the period of gestation in a human female; a question of such importance in relation to the legitimacy of offspring. The period of gestation the author fixes at the 280th day after conception; and he contends that in the human female it is not liable to fluctuation as it is in the females of many classes of animals. (p. 236.) Gestation is limited by the maturity of the child, and when the process advances in an uninterrupted manner, parturition may be expected to commence at the termination of the *fortieth week.* He does not allow that some children are longer in reaching maturity than others; and he argues that maturity is not dependent on the size, weight, or healthiness of the offspring, but on the power of its organs to sustain and carry on an independent existence; an opinion borne out by the fact, that in ordinary gestation the children are as fully developed as in those cases in which gestation has extended beyond the natural period. (p. 253.) He especially cautions us against mistaking *protracted parturition* for *protracted gestation*, a point which has not perhaps by all advocates of the latter doctrine been sufficiently attended to. One case is mentioned by him where parturition commenced as usual at the 280th day. The pains were weak and accompanied by remissions. In this way labour was not completed until after the lapse of fourteen days. (p. 238.) Doubtless a case of this kind would have been set down by many as one of gestation protracted to the 294th day. Some diseases affecting the uterus, more particularly the cervix uteri, may also prevent parturition from being completed at the full time. So again the fœtus may die from the efforts of the uterus at expulsion; and if the membranes be entire, putrefaction is retarded, and the child may be borne for a considerable period, sometimes for many years. Cases of this kind are mentioned, not however from the author's personal observation. (p. 240.) It has been often urged as an argument in favour of protracted gestation, that the frequent premature deliveries which ensue show that the duration of pregnancy is not a fixed term; but the circumstances are different. The premature expulsion of the child is generally to be traced to some injurious influence, acting directly on the uterus or through the system of the mother. There are many causes which operate in this manner, but there are few which tend to delay parturition, with the exception of those already mentioned. The author for these reasons very properly censures that kind of legislation by which the duration of pregnancy is made a fixed term, as in the Code Napoleon. He shrewdly observes, that natural laws are more firmly fixed and more constantly followed than municipal. In investigating a case of this kind, he recommends the practitioner to procure answers to certain questions which he proposes; and of which we shall say they are well adapted to the purpose intended. (p. 250.) We must refer our readers to the volume itself for these.

The second part of the work has reference to the changes induced by parturition, and the state of the parturient female. The eighth chapter is devoted to the details of delivery which he divides into five periods, and which we find treated in a way not differing materially from the rules laid down in most of our standard works on midwifery. In regard to the muscular power of the uterus he observes:

> " We have no rule by which we can measure the force of the expulsive pains, but if I am not mistaken, a powerful man pressing with his whole force against the head of the child at the outlet during delivery would be unable to prevent its expulsion. It is at this period when such extraordinary muscular power is exerted by the uterus, that the bones of the child are liable to be broken." (p. 281.)

In the ninth chapter we have an enquiry as to how far a child may be exposed to danger, from the mother being wholly unacquainted with the course and progress of delivery. " An inexperienced woman," remarks the author, " is not likely to have the power of distinguishing the pains of parturition from those of colic or from pain in the bowels." (p. 293.) "Even if she should imagine delivery had begun, she would not be able to judge how far it had advanced or when it was likely to terminate."

(p. 362.) At that period of delivery where precautions are most necessary to the safety of the child, women are apt to experience a strong desire to evacuate the bowels, and it often requires considerable firmness and resolution on the part of the attendants to prevent them from yielding to this. Again, sometimes delivery is rapid and the pains are not severe; a woman then who believes that the process has not actually commenced, or that it has advanced but little may be unexpectedly delivered while at a water-closet, and the child accidentally destroyed. "These rapid deliveries are sometimes to be ascribed to disorders of the bowels, as colic, cholera, or diarrhœa with tenesmus." (p. 309.) It is acknowledged, however, "that they are not very common among those who become for the first time pregnant." (p. 310.)

We agree with the author, that in doubtful cases when a woman is charged with infanticide, we should make the fullest allowance for her having been unexpectedly delivered, and the child destroyed by falling into the soil of a privy, the mother in her ignorance having mistaken the pains of labour for a desire to evacuate her bowels; but this is so very common and so specious a defence, that we cannot be too much on our guard against being misled by it. Professor Jörg does not seem to imagine that children are ever intentionally destroyed in this manner; but, as medical jurists, we must not shut our eyes to the well known fact, that this is not only a common method of committing murder, but in general a most effectual screen for guilt. Let the medical witness remember, although the circumstance is not even hinted at in the work before us, that accident may now and then enable him to detect a guilty woman. Thus, if the umbilical cord on the body of a child found in such a situation be *cut* or cut and tied, if the head be severed from the body or there be incised wounds about the trunk, such a defence as that suggested by Jörg is of course inadmissible. In one remarkable instance, which occurred a few years since in the west of England, the falsehood of the woman's statement was clearly proved by the medical witness having discovered that the umbilical cord had been cut and tied.

We next find the author laying down the axiom: "That, in general, a female cannot be held responsible for her acts from the commencement of actual labour until the expulsion of the child; and that during the passage of the head through the outlet, the greater number of women are in a state of unconsciousness and ought not to be regarded as free agents." (p. 319.) It is difficult to say how far such a rule should be admitted in practice; for every criminal case must be judged of according to its own merits, but at the same time, when severe injuries are found on a new-born child, due allowance should be made for the temporary loss of consciousness and convulsive action into which extreme pain may plunge a female during parturition. We then come to the important question as to how long such a state may persist *after delivery*, the answer to which must vary according to circumstances. We can undertake to say that, in this country, both medical witnesses as well as courts of law invariably interpret every fact in the most favorable light for the prisoner, to such a degree indeed as to render the serious admonitions of Professor Jörg superfluous to the British medical jurist. There are other causes which, as it is well known, may lead to an attack of delirium or puerperal mania; to these it is unnecessary to advert.

The last chapter is occupied with the *accidental* causes of the death of the child during delivery. Need we say that this part of the subject involves most important questions in relation to charges of infanticide? The attention of the examiner must first be directed to the probable age of the child. It may be immature, in which case its death may have been due to atelectasis, i. e. to a want of proper expansion in the lungs. The characters of the fœtus at different periods of gestation are well given, but present nothing novel. The author is well aware from the researches of Dr. Edward Jörg, that the sinking of the lungs is not a positive proof of a child having been born dead. Another point to consider is, whether there may not be some abnormal condition or want of development in vital organs to account for death. The author believes that a rapid and easy delivery is dangerous to both mother and child, to the latter from its tendency to induce atelectasis. On the other hand, a protracted labour with violent efforts on the part of the uterus, often leads to the death of the child. It is in these cases that the bones of the head become fractured, and that effusion of blood on the brain or cerebral congestion is likely to occur. The author believes on the authority of a case by Roederer, that death may be caused by the cervix uteri embracing the neck of a child. Compression of the umbilical cord is a well known and not unfrequent cause of death. Partial detachment of the placenta or a rupture of the vessels may also prove fatal. When respiration is established before birth, death may take place from suffocation; and the risk of this will be greater in proportion to the length of time which the child remains fixed within the outlet. So a child being born with its membranes enveloping the head, or with its mouth and fauces filled by water or mucus, is thereby rendered unable to respire. Accidental suffocation may also ensue when it is expelled in such a way that its face falls into the discharges of the mother, or becomes closely covered by the clothes. The neglect to apply a ligature to the umbilical cord, or the improper application of it, with the exposure of the child to the air of a cold apartment, is also an occasional cause of death.

We have here merely glanced at these causes, but they are very fairly discussed by the author. Perhaps he is too concise in his remarks on accidental fractures of the cranium during delivery, considering that this is a subject which has only lately called forth particular notice, and one which has an important bearing in cases of alleged child-murder.

We thus complete our review of a work, which, as the reader will perceive from our criticism, is one of somewhat unequal merit. The author sets out with a manifest bias; and, as we have seen, states his object to be that of employing the resources of science to set aside *groundless* charges against females in the pregnant or parturient state. We cannot accuse him of the *suggestio falsi*, but we must certainly charge him in several instances with the *suppressio veri*. If he intended his volume as a guide for a medical witness in these investigations, it is in many respects a signal failure; it is rather a manual expressly adapted for those advocates who undertake the *defence* of females accused of having violated the laws. All the fallacies of medical evidence are put prominently forward; ingenious explanations are invented as substitutes for ordinary medical views; while, on the other hand, scarcely a single fact is produced to enable a medical witness to give that evidence which is necessary to support an accusation. Now, if such crimes as abortion and infanticide had no existence, we would allow the author, however we might differ from him in opinion, the greatest merit for humanity: but abortion and infanticide are unfortunately crimes of too frequent occurrence; and we hold it to be the duty of a medical jurist not to attempt to baffle the law in its endeavours to suppress them, but to assist by bringing forward the light of science to detect and expose them. What should we say of an experienced individual, who, following the example of the author, collected and published only those medical principles which would lead to the conviction of accused parties, carefully withholding the exceptions to these and leaving them to chance-discovery? There might be less humanity on the part of such a person, but it appears to us there would not be greater indiscretion. When Professor Jörg represents himself as using every effort to set aside what he terms *groundless* charges by the rules of science, are we thereby to understand that all such charges are groundless? If not, why has he not brought forward facts to support those which are not groundless? We consider this to be a bad example to medical jurists; they ought, in our estimation, to be wholly independent of the prosecutor and the prosecuted; and while, on the one hand, they vindicate innocence, on the other, they are bound to see that the laws of their country are not lightly violated. We shall conclude our notice by saying that, if we except this error into which we conceive the author has fallen, there are few modern works in which the signs of pregnancy, abortion, and delivery are more ably treated. We object not to the matter of the work so much as to the spirit in which it has been written.

---

